# How to Deprive Iodine of Its Stain

**Published:** 1874-03

**Authors:** 


### Selections.

### HOW TO DEPRIVE IODINE OF ITS STAIN.

Add a few drops of phenic acid to the tincture, and it will not stain; moreover, the tincture is more efficacious and its action more certain. M. Bogs recommends the following formula for use in injections: Alcoholic tincture of iodine, three grm.; phenic acid, six drops.; glycerine, 30 grm.; distilled water, 150 grm. This preparation is superior to all others in the treatment of blennorrhagia and leucorrhœa.—*Med. & Surg. Rep.*

